# Supplementary material for: Improving provider and client communication around family planning in Togo: Results from a cross-sectional survey
Source: PLOS Glob Public Health. 2023 Jun 8;3(6):e0001923. doi: 10.1371/journal.pgph.0001923 (PMC10249824; doi:10.1371/journal.pgph.0001923)
Supplement: S1 File — (DOCX) [file pgph.0001923.s001.docx]

S1 File: Full regression output

**Outcome 1: Complete FP history**

xtmelogit complete_history_all group age25_34 age35plus primaryless priv_confid status_notusing status_switch number_staff || q001:

Refining starting values:

Iteration 0: log likelihood = -409.28739

Iteration 1: log likelihood = -405.60323

Iteration 2: log likelihood = -405.29163

Performing gradient-based optimization:

Iteration 0: log likelihood = -405.29163

Iteration 1: log likelihood = -405.28735

Iteration 2: log likelihood = -405.28735

Mixed-effects logistic regression Number of obs = 885

Group variable: q001 Number of groups = 25

Obs per group:

min = 1

avg = 35.4

max = 71

Integration points = 7 Wald chi2(8) = 47.40

Log likelihood = -405.28735 Prob > chi2 = 0.0000

---------------------------------------------------------------------------------

complete_hist~l | Coef. Std. Err. z P>|z| [95% Conf. Interval]

----------------+----------------------------------------------------------------

group | 1.595415 .7856415 2.03 0.042 .0555863 3.135244

age25_34 | -.1209904 .20367 -0.59 0.552 -.5201763 .2781955

age35plus | -.4384833 .253045 -1.73 0.083 -.9344424 .0574757

primaryless | .1895918 .1981103 0.96 0.339 -.1986972 .5778808

priv_confid | .3244954 .261971 1.24 0.215 -.1889584 .8379493

status_notusing | 1.173401 .2320283 5.06 0.000 .7186337 1.628168

status_switch | .9832531 .2496431 3.94 0.000 .4939617 1.472545

number_staff | -.0724992 .0289362 -2.51 0.012 -.1292132 -.0157852

_cons | -1.643683 .7361359 -2.23 0.026 -3.086483 -.2008836

---------------------------------------------------------------------------------

------------------------------------------------------------------------------

Random-effects Parameters | Estimate Std. Err. [95% Conf. Interval]

-----------------------------+------------------------------------------------

q001: Identity |

sd(_cons) | 1.561323 .3245647 1.038836 2.346599

------------------------------------------------------------------------------

LR test vs. logistic model: chibar2(01) = 108.38 Prob >= chibar2 = 0.0000

xtmelogit, or

Mixed-effects logistic regression Number of obs = 885

Group variable: q001 Number of groups = 25

Obs per group:

min = 1

avg = 35.4

max = 71

Integration points = 7 Wald chi2(8) = 47.40

Log likelihood = -405.28735 Prob > chi2 = 0.0000

---------------------------------------------------------------------------------

complete_hist~l | Odds Ratio Std. Err. z P>|z| [95% Conf. Interval]

----------------+----------------------------------------------------------------

group | 4.930377 3.873509 2.03 0.042 1.05716 22.99426

age25_34 | .8860425 .1804603 -0.59 0.552 .5944157 1.320744

age35plus | .645014 .1632175 -1.73 0.083 .3928049 1.05916

primaryless | 1.208756 .239467 0.96 0.339 .8197981 1.782258

priv_confid | 1.383333 .3623931 1.24 0.215 .827821 2.311622

status_notusing | 3.232968 .7501401 5.06 0.000 2.051628 5.094532

status_switch | 2.673138 .6673304 3.94 0.000 1.638796 4.360316

number_staff | .9300665 .0269126 -2.51 0.012 .8787866 .9843388

_cons | .1932669 .1422707 -2.23 0.026 .0456623 .8180077

---------------------------------------------------------------------------------

Note: _cons estimates baseline odds (conditional on zero random effects).

------------------------------------------------------------------------------

Random-effects Parameters | Estimate Std. Err. [95% Conf. Interval]

-----------------------------+------------------------------------------------

q001: Identity |

sd(_cons) | 1.561323 .3245647 1.038836 2.346599

------------------------------------------------------------------------------

LR test vs. logistic model: chibar2(01) = 108.38 Prob >= chibar2 = 0.0000

**Outcome 2: MIIplus**

xtmelogit miiplus_yes group age25_34 age35plus primaryless priv_confid status_notusing status_switch number_staff || q001:

Refining starting values:

Iteration 0: log likelihood = -444.9476

Iteration 1: log likelihood = -442.59526

Iteration 2: log likelihood = -442.58159

Performing gradient-based optimization:

Iteration 0: log likelihood = -442.58159

Iteration 1: log likelihood = -442.58154

Iteration 2: log likelihood = -442.58154

Mixed-effects logistic regression Number of obs = 885

Group variable: q001 Number of groups = 25

Obs per group:

min = 1

avg = 35.4

max = 71

Integration points = 7 Wald chi2(8) = 129.77

Log likelihood = -442.58154 Prob > chi2 = 0.0000

---------------------------------------------------------------------------------

miiplus_yes | Coef. Std. Err. z P>|z| [95% Conf. Interval]

----------------+----------------------------------------------------------------

group | 1.3677 .5371535 2.55 0.011 .3148982 2.420501

age25_34 | .3312492 .1970324 1.68 0.093 -.0549273 .7174257

age35plus | .0476474 .2284453 0.21 0.835 -.4000971 .4953919

primaryless | -.1940702 .1815555 -1.07 0.285 -.5499124 .161772

priv_confid | 1.257163 .2297339 5.47 0.000 .8068928 1.707433

status_notusing | 1.795074 .227965 7.87 0.000 1.348271 2.241877

status_switch | 1.980815 .2445898 8.10 0.000 1.501428 2.460202

number_staff | -.0212375 .0192112 -1.11 0.269 -.0588908 .0164158

_cons | -2.082919 .5474087 -3.81 0.000 -3.15582 -1.010018

---------------------------------------------------------------------------------

------------------------------------------------------------------------------

Random-effects Parameters | Estimate Std. Err. [95% Conf. Interval]

-----------------------------+------------------------------------------------

q001: Identity |

sd(_cons) | 1.011064 .2095215 .6735743 1.517652

------------------------------------------------------------------------------

LR test vs. logistic model: chibar2(01) = 59.33 Prob >= chibar2 = 0.0000

.

. xtmelogit, or

Mixed-effects logistic regression Number of obs = 885

Group variable: q001 Number of groups = 25

Obs per group:

min = 1

avg = 35.4

max = 71

Integration points = 7 Wald chi2(8) = 129.77

Log likelihood = -442.58154 Prob > chi2 = 0.0000

---------------------------------------------------------------------------------

miiplus_yes | Odds Ratio Std. Err. z P>|z| [95% Conf. Interval]

----------------+----------------------------------------------------------------

group | 3.926309 2.10903 2.55 0.011 1.37012 11.2515

age25_34 | 1.392707 .2744084 1.68 0.093 .9465539 2.049151

age35plus | 1.048801 .2395936 0.21 0.835 .670255 1.641141

primaryless | .8236001 .1495291 -1.07 0.285 .5770003 1.175592

priv_confid | 3.515434 .8076141 5.47 0.000 2.240934 5.514787

status_notusing | 6.01992 1.372331 7.87 0.000 3.850761 9.41098

status_switch | 7.248649 1.772946 8.10 0.000 4.488092 11.70718

number_staff | .9789865 .0188075 -1.11 0.269 .9428097 1.016551

_cons | .1245661 .0681886 -3.81 0.000 .0426034 .3642126

---------------------------------------------------------------------------------

Note: _cons estimates baseline odds (conditional on zero random effects).

------------------------------------------------------------------------------

Random-effects Parameters | Estimate Std. Err. [95% Conf. Interval]

-----------------------------+------------------------------------------------

q001: Identity |

sd(_cons) | 1.011064 .2095215 .6735743 1.517652

------------------------------------------------------------------------------

LR test vs. logistic model: chibar2(01) = 59.33 Prob >= chibar2 = 0.0000

**Outcome 3: STI/HIV risk**

xtmelogit partner_all2 group age25_34 age35plus primaryless priv_confid status_notusing status_switch number_staff || q001:

Refining starting values:

Iteration 0: log likelihood = -295.95716

Iteration 1: log likelihood = -292.99499

Iteration 2: log likelihood = -292.76277

Performing gradient-based optimization:

Iteration 0: log likelihood = -292.76277

Iteration 1: log likelihood = -292.75923

Iteration 2: log likelihood = -292.75923

Mixed-effects logistic regression Number of obs = 885

Group variable: q001 Number of groups = 25

Obs per group:

min = 1

avg = 35.4

max = 71

Integration points = 7 Wald chi2(8) = 48.50

Log likelihood = -292.75923 Prob > chi2 = 0.0000

---------------------------------------------------------------------------------

partner_all2 | Coef. Std. Err. z P>|z| [95% Conf. Interval]

----------------+----------------------------------------------------------------

group | 1.981237 .8595226 2.31 0.021 .2966039 3.665871

age25_34 | -.4259716 .2387336 -1.78 0.074 -.8938809 .0419376

age35plus | -.1714163 .307873 -0.56 0.578 -.7748362 .4320036

primaryless | -.2142283 .2426822 -0.88 0.377 -.6898767 .2614201

priv_confid | 1.326218 .4149841 3.20 0.001 .5128639 2.139572

status_notusing | 1.212527 .2718797 4.46 0.000 .6796529 1.745402

status_switch | 1.074573 .2878943 3.73 0.000 .5103102 1.638835

number_staff | -.061555 .031782 -1.94 0.053 -.1238465 .0007366

_cons | -4.173468 .9308533 -4.48 0.000 -5.997907 -2.349029

---------------------------------------------------------------------------------

------------------------------------------------------------------------------

Random-effects Parameters | Estimate Std. Err. [95% Conf. Interval]

-----------------------------+------------------------------------------------

q001: Identity |

sd(_cons) | 1.575078 .3892634 .9703691 2.556627

------------------------------------------------------------------------------

LR test vs. logistic model: chibar2(01) = 56.27 Prob >= chibar2 = 0.0000

.

. xtmelogit, or

Mixed-effects logistic regression Number of obs = 885

Group variable: q001 Number of groups = 25

Obs per group:

min = 1

avg = 35.4

max = 71

Integration points = 7 Wald chi2(8) = 48.50

Log likelihood = -292.75923 Prob > chi2 = 0.0000

---------------------------------------------------------------------------------

partner_all2 | Odds Ratio Std. Err. z P>|z| [95% Conf. Interval]

----------------+----------------------------------------------------------------

group | 7.25171 6.233009 2.31 0.021 1.345282 39.09016

age25_34 | .6531349 .1559252 -1.78 0.074 .4090651 1.042829

age35plus | .8424708 .259374 -0.56 0.578 .4607792 1.540341

primaryless | .8071641 .1958844 -0.88 0.377 .5016379 1.298773

priv_confid | 3.76677 1.563149 3.20 0.001 1.670067 8.495798

status_notusing | 3.361971 .9140515 4.46 0.000 1.973193 5.728202

status_switch | 2.928741 .8431677 3.73 0.000 1.665808 5.149167

number_staff | .9403012 .0298846 -1.94 0.053 .8835154 1.000737

_cons | .0153988 .014334 -4.48 0.000 .0024839 .0954618

---------------------------------------------------------------------------------

Note: _cons estimates baseline odds (conditional on zero random effects).

------------------------------------------------------------------------------

Random-effects Parameters | Estimate Std. Err. [95% Conf. Interval]

-----------------------------+------------------------------------------------

q001: Identity |

sd(_cons) | 1.575078 .3892634 .9703691 2.556627

------------------------------------------------------------------------------

LR test vs. logistic model: chibar2(01) = 56.27 Prob >= chibar2 = 0.0000
