# Supplementary material for: Improving provider and client communication around family planning in Togo: Results from a cross-sectional survey
Source: PLOS Glob Public Health. 2023 Jun 8;3(6):e0001923. doi: 10.1371/journal.pgph.0001923 (PMC10249824; doi:10.1371/journal.pgph.0001923)
Supplement: Attachment — Submitted filename: PlosResponseToReviewers_10Apr2023.docx [file pgph.0001923.s002.docx]

**RESPONSE TO REVIEWERS**

Reviewer #1: This is an interesting and well written manuscript. I do have some questions and suggestions for your consideration.

1. I see that you have conducted post-test power calculations. Did you also conduct pre-test power calculations? If not, is there a reason why not, and if so, is there a reason why you chose to present the post-test calculations instead? In that same vein, can you explain why the client volume in the control facilities was so much lower than expected.

*The original study design was a pre-post with comparison w**ith an estimated sample size of approximately 600 female FP clients in the intervention area and approximately 400 clients in the comparison area (total N=1,000) per round. This pre-test power calculation was based on a previous evaluation assessing client satisfaction, this sample size is based on a minimum detectable difference of 14 percent in the priority indicator between study groups, average score on the validated Quality of Care scales, with 80% power to detect a difference and alpha of 0.05 (32) and assuming a narrow distribution of scores. Based on sampling guidance from the World Health Organizations, Service Availability and Readiness Assessment (SARA) implementation guide which applies a design effect of 1.2 to account for intracluster correlation, we inflate the design effect to 1.5 to account for higher intracluster correlation due to a smaller number of facilities in our sample as compared to the number of facilities included in the SARA (33). Although a wide range of specific indicators were measured and assessed quantitatively in the study, this indicator was selected to power the study evaluating effectiveness over time in the study population. We have added text to the methods to explain that we originally conducted a pre-test power calculations (lines 102-106 on tracked changes version).*

*In terms of why client volume was low at the control facilities, we did not identify a reason why this occurred. The qualitative data did not give us any indication.*

2. Can you please elaborate on the intervention and how it relates to the outcomes you were measuring. Can you also please describe when the intervention occurred in relation to the survey? Was the survey immediately after the intervention, or had some time passed, which might suggest sustainability of results? Also, you state in the discussion that the providers have welcomed the interrelated elements of the intervention. Did you collect quantitative or qualitative feedback from providers to assess their satisfaction with the intervention?

*We included dates for the provider communication training (lines 123-125). The provider communication training was directly relevant to the study outcomes. This has been made explicit in the text (lines 150-151).*

*We collected qualitative feedback from providers to assess their satisfaction with the intervention. Findings will be available in the forthcoming manuscript which is currently under review Dougherty L. et al.* *A qualitative exploration of how a social accountability approach influences community and health worker perceptions related to family planning service delivery in Togo. BMC Health Services Research. 2023. We have added this reference to the publication list.*

3. Was there a reason why you conducted both observations of client visits and client exit interviews? If you were observing visits, could MII and MIIplus have been collected during those visits instead of at a separate CEI?

*Client-provider observations were conducted to assess process attributes including interpersonal communication, and FP counseling provided by health care providers to clients and to assess the association of intervention exposure and provider interpersonal communication and FP counseling. Client exit interviews were conducted to assess client satisfaction and other measures from the client’s perspective. We have added text to the methods section to clarify this (lines 111-113). Client exit interviews were used for the Method Information Index measures since these metrics are typically assessed from the contraceptive user’s perspective.*

4. Depending on how it is defined, "ensuring privacy" could be either a structural quality component or a process quality component. I think that you are considering privacy to be a structural quality component, but it would be helpful to describe how you are defining privacy in this context.

*Privacy in this context is structural and refers to an environment that where both visual and auditory privacy of clients is ensured. This clarification has been added to the text (lines 140-141).*

5. You allude to this in the conclusion, but the ultimate goal of improved provider communication is improved client satisfaction, higher FP uptake/continuation, or both. Did you measure either of those outcomes? Are they correlated with the improved communication that you observed at intervention facilities?

*We measured client satisfaction, but almost all clients reported being satisfied with their FP visit. As such, these variables were not useful for analysis. We have now included this in the methods (lines 102-106) section and refer to it in the discussion section (lines 233-236).*

Reviewer #2: General comments

The manuscript addresses an important and sometimes neglected issue namely early childhood caries. The findings of an in-depth scoping review focusing on the Gulf Cooperation Council (GCC) countries is resented. While the summary of the current knowledge reported is compelling, there are concerns in the way the way the evidence is presented.

*This first summary set of comments is confusing to us since this article is not focused on early childhood caries or in the Gulf Cooperation Council countries. We believe there has been an error in copying over this comment for this review.*

Specific Comments
Title – The title could be improved to include the study design used to answer the research question – cross sectional survey (as described in the methods). The portion “Results from an intervention in Togo” is not very informative.

*The title has been changed to “*Improving provider and client communication around family planning in Togo: Results from a cross-sectional survey”

Abstract – The abstract is well written and summarizes the rationale for the study, methodology used, key findings, and conclusions. However, the key findings could be strengthened by including some of the significant results from the multivariate analysis i.e. ORs, p-values, etc.

*Due to word count constraints (300 words), we were unable to add substantial details to the abstract. We did, however, include the p-value (p<0.05) for the multivariate results.*

Introduction - The authors have cited relevant studies and provided good background, focusing on the main focus of the manuscript.

Materials and Methods –

- The paragraph on ethical approval seems oddly phrased for a manuscript, e.g. remove language referencing the editorial office, among other things --- rephrase. “The relevant ethical approval and consent details were received and are available on request by the editor or editorial office. Study participants provided informed consent by using their signature. In addition, all methods were carried out in accordance with relevant guidelines and regulations and with the 1964 Helsinki declaration and its later amendments or comparable ethical standards.”

*We have deleted these sentences.*

- Outcome variables and the analysis conducted are well defined.

Results – Summary table presented is well laid out and good accompanying text highlighting the key findings.

Discussion/ Conclusion – These two sections frame the findings well in answering the research question.

References – Relevant and recent.

Other comments – Minor editing required; authors to review the entire manuscript and make the relevant changes. For example, abbreviation MOH first appears on line 76, then it’s defined later in line 238. This should be the other way round.

*Thank you for catching this. We have made this change along with additional edits throughout.*
